# Reliability of a Smartphone App to Objectively Monitor Performance Outcomes in Degenerative Cervical Myelopathy: Observational Study

**DOI:** 10.2196/56889

**Published:** 2024-05-24

**Authors:** Alvaro Yanez Touzet, Tatiana Houhou, Zerina Rahic, Angelos Kolias, Stefan Yordanov, David B Anderson, Ilya Laufer, Maggie Li, Gordan Grahovac, Mark RN Kotter, Benjamin M Davies, MoveMed Ltd

**Affiliations:** 1 School of Medical Sciences Faculty of Biology, Medicine and Health University of Manchester Manchester United Kingdom; 2 MoveMed Ltd. Cambridge United Kingdom; 3 Division of Science New York University Abu Dhabi Abu Dhabi United Arab Emirates; 4 Division of Neurosurgery Addenbrooke's Hospital University of Cambridge Cambridge United Kingdom; 5 ANAPLASI Rehabilitation Centre Athens Greece; 6 1st Neurosurgery Department Henry Dunant Hospital Center Athens Greece; 7 Faculty of Medicine and Health Sydney School of Health Sciences The University of Sydney Sydney Australia; 8 New York University Langone Health New York, NY United States; 9 King's College Hospital Kings NHS Foundation Trust Denmark Hill London United Kingdom; 10 Department of Clinical Neurosurgery University of Cambridge Cambridge United Kingdom; 11 Department of Clinical Neurosciences Ann McLaron Laboratory of Regenerative Medicine Cambridge United Kingdom; 12 Department of Clinical Neurosciences University of Cambridge Cambridge United Kingdom; 13 See Acknowledgements

**Keywords:** reproducibility of results, patient outcome assessment, smartphone, neurology, psychometrics, spinal cord compression, mobile phone

## Abstract

**Background:**

Developing new clinical measures for degenerative cervical myelopathy (DCM) is an AO Spine RECODE-DCM Research, an international and multi-stakeholder partnership, priority. Difficulties in detecting DCM and its changes cause diagnostic and treatment delays in clinical settings and heightened costs in clinical trials due to elevated recruitment targets. Digital outcome measures can tackle these challenges due to their ability to measure disease remotely, repeatedly, and more economically.

**Objective:**

The aim of this study is to assess the reliability of the MoveMed battery of performance outcome measures.

**Methods:**

A prospective observational study in decentralized secondary care was performed in England, United Kingdom. The primary outcome was to determine the test-retest reliability of the MoveMed performance outcomes using the intraclass correlation (ICC) of agreement . The secondary outcome was to determine the measurement error of the MoveMed performance outcomes using both the SE of the mean (SEM) of agreement and the smallest detectable change (SDC) of agreement . Criteria from the Consensus-Based Standards for the Selection of Health Measurement Instruments (COSMIN) manual were used to determine adequate reliability (ie, ICC of agreement ≥0.7) and risk of bias. Disease stability was controlled using 2 minimum clinically important difference (MCID) thresholds obtained from the literature on the patient-derived modified Japanese Orthopaedic Association (p-mJOA) score, namely, MCID ≤1 point and MCID ≤2 points.

**Results:**

In total, 7 adults aged 59.5 (SD 12.4) years who live with DCM and possess an approved smartphone participated in the study. All tests demonstrated moderate to excellent test-retest coefficients and low measurement errors. In the MCID ≤1 group, ICC of agreement values were 0.84-0.94 in the fast tap test, 0.89-0.95 in the hold test, 0.95 in the typing test, and 0.98 in the stand and walk test. SEM of agreement values were ±1 tap, ±1%-3% stability score points, ±0.06 keys per second, and ±10 steps per minute, respectively. SDC of agreement values were ±3 taps, ±4%-7% stability score points, ±0.2 keys per second, and ±27 steps per minute, respectively. In the MCID ≤2 group, ICC of agreement values were 0.61-0.91, 0.75-0.77, 0.98, and 0.62, respectively; SEM of agreement values were ±1 tap, ±2%-4% stability score points, ±0.06 keys per second, and ±10 steps per minute, respectively; and SDC of agreement values were ±3-7 taps, ±7%-10% stability score points, ±0.2 keys per second, and ±27 steps per minute, respectively. Furthermore, the fast tap, hold, and typing tests obtained sufficient ratings (ICC of agreement ≥0.7) in both MCID ≤1 and MCID ≤2 groups. No risk of bias factors from the COSMIN Risk of Bias checklist were recorded.

**Conclusions:**

The criteria from COSMIN provide “very good” quality evidence of the reliability of the MoveMed tests in an adult population living with DCM.

## Introduction

Degenerative cervical myelopathy (DCM) is a slow-motion spinal cord injury that is estimated to affect 1 in 50 adults [1,2]. In this disabling condition, dexterity, gait, and balance are key measurement constructs. As with numerous other diseases that affect the nervous and musculoskeletal systems, the monitoring of DCM today still largely depends on qualitative approaches such as hierarchical classifications of exemplar functions. While such measures can be robust, they remain intrinsically subjective. Hence, such measures pose significant challenges, especially in fluctuating diseases, as they are often unable to effectively detect small but significant changes in a timely fashion.

The current gold standard measure in DCM is the modified Japanese Orthopaedic Association (mJOA) score, but, unfortunately, it is found to possess low and inadequate responsiveness to detect disease change [3,4]. Variation, driven by both disease heterogeneity and instrument reliability, is more than twice the minimum clinically important difference (MCID). In practice, this demands sample sizes greater than 300 patients for 1:1 comparison with at least 80% power. Developing new approaches to functional measurement is, thus, a recognized research priority in DCM. Quantitative, quick, and decentralized disease monitoring could significantly improve control of heterogeneity and critical sample sizes, for instance, through the use of performance-based outcomes (PerfOs) and performance-based outcome measures (PerfOMs).

The MoveMed app (MoveMed Ltd), a battery of PerfOMs in a mobile app, recently demonstrated validity in DCM [5]. The app leverages the accuracy of mobile sensors to assess hand, arm, and leg function in real time, in the user’s natural environment, and under standardized conditions. It was designed by health care professionals from the University of Cambridge and is being developed in accordance with ISO (International Organization for Standardization) 13485 (software as a medical device). Its reliability, however, has not yet been formally assessed. Thus, in this study, we use formal methods and criteria from the US Food and Drug Administration (FDA) and Consensus-Based Standards for the Selection of Health Measurement Instruments (COSMIN) to assess the reliability of MoveMed’s PerfOMs. This is the second paper in a series of clinimetric studies on the measurement properties of MoveMed’s PerfOMs [5].

## Methods

### Participants

**Figure 1:**
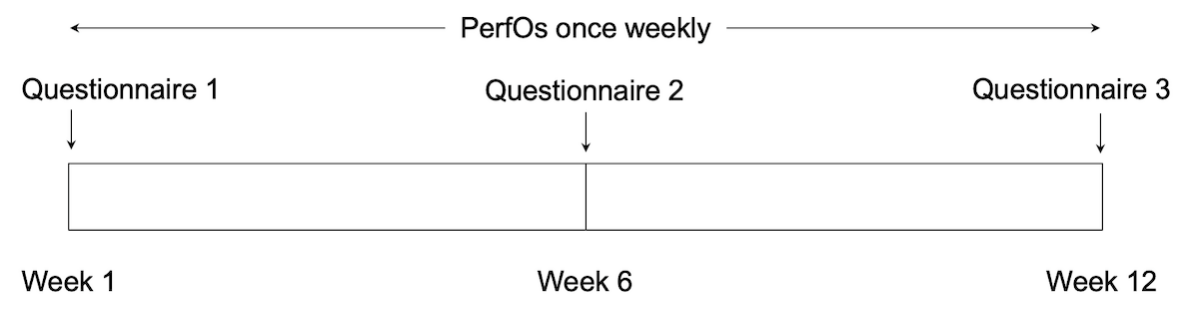
Study timeline. People living with degenerative cervical myelopathy enrolled in a prospective observational study in a decentralized secondary care setting in England, United Kingdom. Over a period of 12 weeks, participants were asked to conduct PerfOs once weekly in the MoveMed app and patient-reported questionnaires every 3 weeks on a web form. PerfO: performance-based outcome.

Between September 2022 and April 2023, people living with a neurological condition were enrolled in the prospective and decentralized EMPOWER study. Prospective participants were recruited via a web-based campaign and asked to complete consent and registration forms (Figure 1) [6,7]. These were used to screen participants for eligibility. Participants were deemed eligible if they had a self-reported diagnosis of DCM, owned a smartphone, and were able to stand and walk without the assistance of another person. Eligible participants were invited to download the MoveMed app to their smartphones and complete an electronic, baseline questionnaire on neuromuscular function, hand dominance, and quality of life (QOL). This included questions from the patient-derived modified Japanese Orthopaedic Association (P-mJOA) and the World Health Organization Quality of Life Brief Version (WHOQOL-Bref) scores.

All enrolled participants were asked to perform each task in the MoveMed app once per week for a period of 12 weeks. Task adherence was remotely monitored once a week using a bespoke web-based dashboard. Participants were offered reminders and help via email once a week if 14 days passed since the completion of the latest task. These were offered for a total of 2 consecutive times per participant, after which the participant was considered lost to follow-up. At weeks 6 and 12, participants were asked to complete the same electronic questionnaire from week 1.

### MoveMed and Tasks

**Figure 2:**
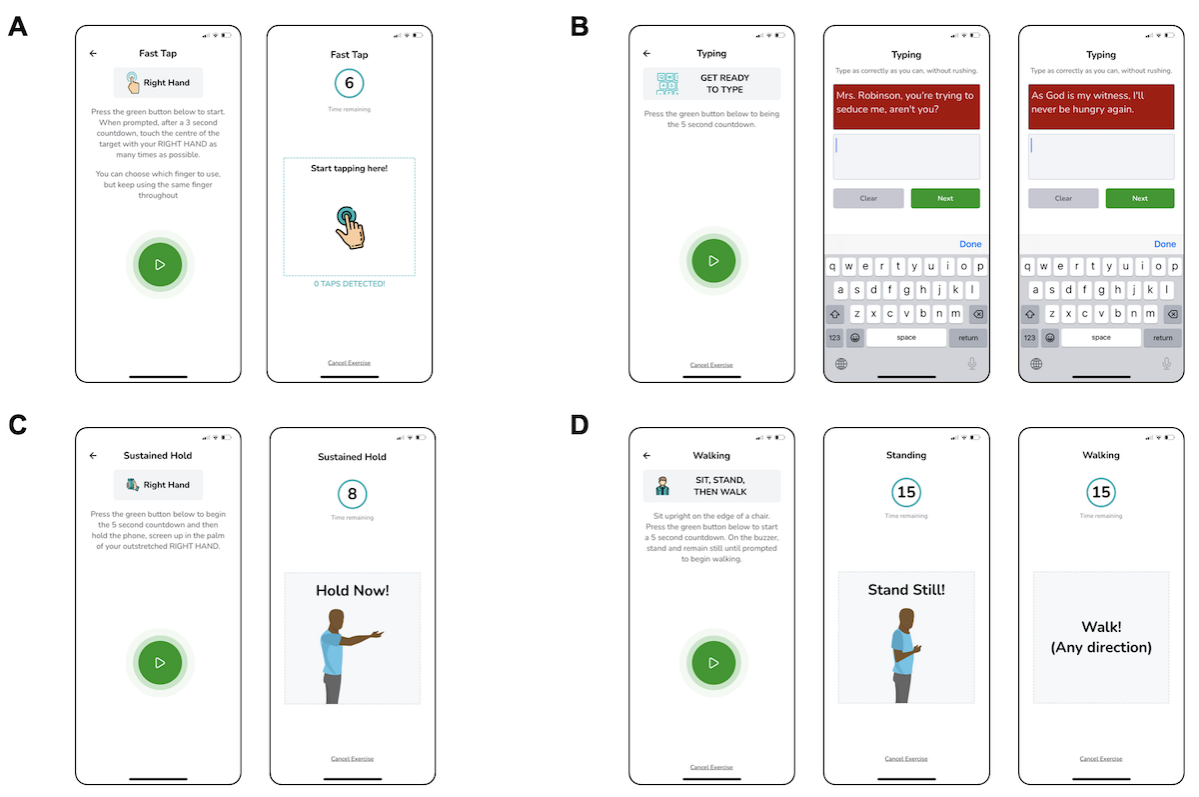
Schematic illustrations of the MoveMed battery of performance outcome measures. (A) The 6-second fast tap test, (B) the 2-stage typing test, (C) the 8-second sustain-hold test, and (D) the 15-second stand and walk test.

The MoveMed app is a smartphone app designed by academic neurosurgeons and computer scientists from the University of Cambridge to administer PerfOMs (Figure 2). These may be administered by clinicians during in-person visits or self-performed by individuals in the community. Version 1.0.0 of the app originally offered 3 performance tasks: a fast tap test, a hold test, and a stand and walk test. Version 1.2.2 incorporated an additional offering—a typing test—while making no changes to the 3 original tasks. Versions 1.0.0 and 1.2.2 were, respectively, available in the Android Google Play Store and iOS App Store at the time of writing and were used in this study by enrolled participants.

The fast tap test is a PerfO task that assesses finger dexterity through a 6-second smartphone touch-based task. Users are shown a demonstrative cartoon (Figure 2A) and instructed to “touch the centre of the target with [each] hand as many times as possible.” In-app video demonstration is also available. The construct (finger dexterity) is assessed by measuring the speed, accuracy, and efficiency of finger tapping as continuous variables and analyzing them as a panel of measures. Content and construct validity were previously assessed by Yanez Touzet et al [5]. In this study, tap count and latency were used as reflective measures of finger dexterity.

The typing test is another PerfO task that assesses finger dexterity through a 2-stage smartphone touch-based task. Users are shown a demonstrative cartoon (Figure 2B) and instructed to “type as correctly as they can, without rushing.” In-app video demonstration is also available. The construct (finger dexterity) is assessed by measuring the speed, accuracy, and efficiency of typing as continuous variables and analyzing them as a panel of unidimensional measures. Content and construct validity were previously assessed by Yanez Touzet et al [5]. In this study, typing speed was used as a reflective measure of finger dexterity.

The hold test is a PerfO task that assesses upper limb stability through an 8-second in-hand smartphone task. Users are shown a demonstrative cartoon (Figure 2C) and instructed to “hold the phone, screen up in the palm of [their] outstretched hand.” In-app video demonstration is also available. The construct (upper limb stability) is assessed by measuring the involuntariness, rhythmicity, and oscillation of the upper limbs as continuous variables and analyzing them as a multidimensional stability score. Content and construct validity were previously assessed by Yanez Touzet et al [5]. In this study, the stability score was used as a reflective measure of upper limb stability [5].

The stand and walk test is a PerfO task that assesses gait through a 2-stage in-hand smartphone task. During the first stage, users are instructed to “sit upright on the edge of a chair [and to] press the green button [when they are ready to] stand and remain still.” During the second stage, users are instructed to “walk [in] any direction.” In-app cartoon and video demonstration are also available (Figure 2D). The construct (gait) may then be assessed by measuring standing or walking as continuous variables and analyzing them as multidimensional measures. Content and construct validity were previously assessed by Yanez Touzet et al [5]. In this study, cadence was used as a reflective measure of gait.

### Patient-Reported Outcomes

Two patient-reported outcome measures (PROMs) were used as descriptors for DCM: the P-mJOA and the WHOQOL-Bref. The P-mJOA score is a patient-reported questionnaire that assesses neuromuscular function in DCM across 4 items: motor dysfunction of the upper extremity, motor dysfunction of the lower extremity (MDLE), sensory dysfunction of the upper extremities, and sphincter dysfunction [8]. Responses are scored on an ordinal scale per item and presented as both a panel of scores and an unweighted sum total score. The P-mJOA was selected due to the existence of a systematic assessment of construct validity (*r*>0.5) and feasibility in DCM [3] and due to the use of its clinically reported analog as the current gold standard. The P-mJOA was favored over the mJOA since it is intended to be a truly patient-reported equivalent of the mJOA, which can be understood by individuals with no medical knowledge or training [9].

The WHOQOL-Bref is a patient-reported questionnaire that assesses QOL across 26 items grouped into 4 domains: physical health, psychological health, social relationship, and environmental health [10]. Responses are scored on a 5-point ordinal scale per item and presented as a panel of sum total scores. Responses to 2 items may, furthermore, be presented individually to give insight into the respondent’s global perception of their QOL and their quality of health. These were presented in writing to describe the population’s characteristics but were not considered robust enough to warrant correlation analysis. The WHOQOL-Bref was selected due to the existence of systematic assessments of validity, reliability, and responsiveness in traumatic brain injury [11], Parkinson disease [12], and DCM [3]. It was also favored over the 36-Item Short Form Health Survey due to its relative brevity and over the EuroQol 5-dimensions questionnaire due to licensing restrictions.

### Reliability and Measurement Error

The COSMIN manual defines reliability as “the degree to which scores for patients who have not changed are the same for repeated measurement under several conditions” [13]. Reliability may be formally assessed through test-retest reliability and measurement error.

In this study, reliability was assessed using test-retest reliability as the primary outcome and measurement error as a secondary outcome. Intraclass correlation (ICC) of agreement was computed to assess the test-retest reliability of the PerfO’s continuous results. The SE of the mean (SEM) of agreement and the smallest detectable change (SDC) of agreement were subsequently computed to assess the measurement error of the PerfO’s continuous results. SDC of agreement was computed using the formula 1.96×√2×SEM of agreement [14].

Data from the 12 weeks of the study were included due to the repeated-measures nature of the reliability analysis. Individuals were considered eligible for analysis if (1) there were at least 2 test repeats per PerfO, (2) their P-mJOA scores at weeks 6 and 12 did not change clinically, and (3) there were no contextual anomalies in the time series for that individual, that is, no subset of data points within the time series deviated significantly from the entire data set [15]. The clinical change was assessed using the MCID of the P-mJOA, that is, “between 1 point and 2 points” from baseline, as in Tetreault et al [16]. This meant that P-mJOA scores above MCID at weeks 6 or 12 were considered clinically important and thus ineligible for analysis, while scores below MCID were considered clinically unimportant and thus eligible for analysis. Since the MCID is a range, analyses were subgrouped into the upper limit of the range (ie, an MCID ≤2 subgroup) and a lower limit of the range (ie, an MCID ≤1 subgroup). This was done to aid with the clinical interpretation of the findings, as MCID in DCM varies with disease severity.

All data were analyzed using Python software (version 3.10.12; Python Software Foundation). ICC of agreement coefficients were calculated for successive repeats of the MoveMed PerfOMs using the single random rater model from the *pingouin* package [17]. Missing data were not imputed; thus, time periods with the least missing data were selected for each PerfOM to mitigate the impact of missing data [17]. SEM of agreement and SDC of agreement were calculated using the same series of successive repeats as the ICC of agreement coefficients.

### Risk of Bias Assessment

The COSMIN Risk of Bias checklist [13] was used to assess the methodological quality of test-retest reliability and measurement error.

### Overall Assessment

Overall assessments of reliability were made using a panel of ratings. As in COSMIN [13], ICC of agreement was converted into ratings by comparing results to a critical threshold of 0.7. ICC of agreement ≥0.7 was rated “sufficient.” ICC of agreement <0.7 was rated “insufficient.”

### Ethical Considerations

This study was independently assessed and approved by the University of Cambridge (HBREC.2022.13). All study participants provided informed consent before enrolling in the study and were able to opt out at any point. Study data were anonymized. None of the participants received any form of compensation for enrolling in or completing the trial.

## Results

### Participants

**Figure 3:**
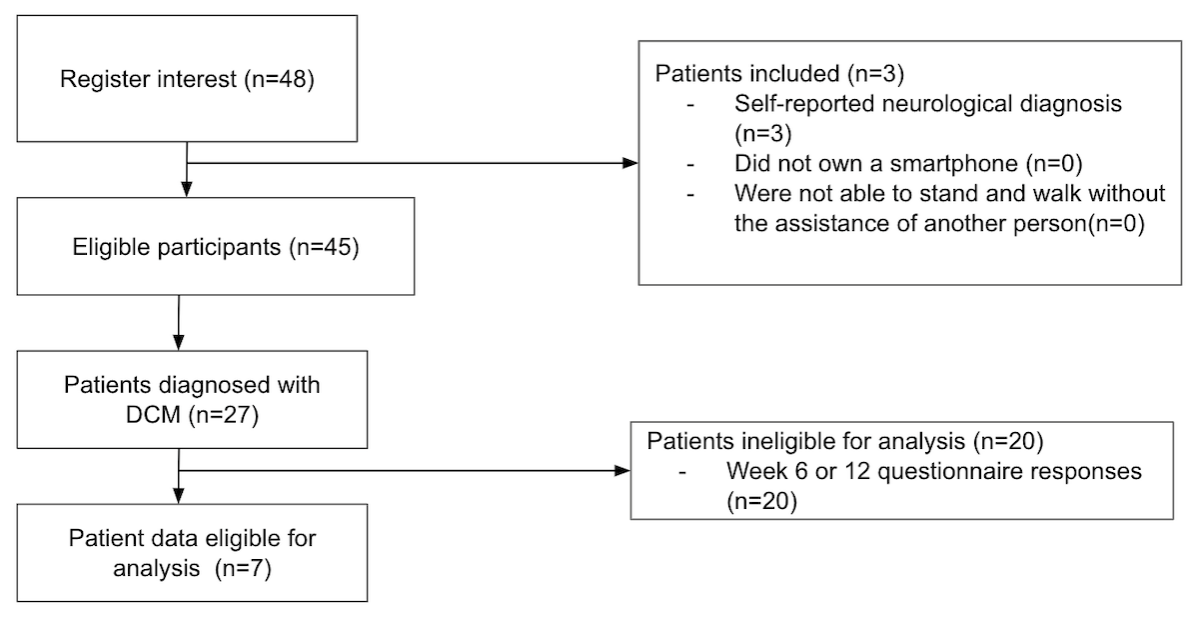
STROBE (Strengthening the Reporting of Observational Studies in Epidemiology) diagram. DCM: degenerative cervical myelopathy.

In total, 27 participants with DCM enrolled in the prospective and decentralized EMPOWER study (Figure 3) principally via advertisement through Myelopathy.org, a DCM charity [6,7]. Of these, 20 (74%) participants were deemed ineligible for analysis due to the absence of a completed follow-up questionnaire (Figure 3), and 7 (26%) met all criteria for analysis (Table 1). From these, 7/7 (100%) were assigned to the MCID ≤2 group and 5/7 (71%) to the more conservative MCID ≤1 group.

**Table 1:** Characteristics of clinically stable study participants (n=7).^a^

Feature		MCID^b^ ≤1	MCID ≤2
Participants, n (%)		5 (71)	7 (100)
Age (years), mean (SD)		62.0 (18.4)	56.5 (12.4)
**P-mJOA^c^ (reference range: 0-18), mean (SD)**		10.8 (2.3)	12.5 (3.6)
	MDUE^d^ (reference range: 0-5)	3.4 (1.1)	3.4 (1.1)
	MDLE^e^ (reference range: 0-7)	3.4 (0.9)	4.1 (1.6)
	SDUE^f^ (reference range: 0-3)	2.0 (0.7)	2.1 (0.8)
	Sphincter dysfunction (reference range: 0-3)	2.0 (0.0)	2.4 (0.5)
**WHOQOL-Bref^g^, mean (SD)**			
	Overall QOL^h^ (reference range: 1-5)	2.6 (0.9)	3.1 (1.0)
	Overall health (reference range: 1-5)	2.6 (0.5)	3.0 (0.8)
	EH^i^ (reference range: 8-40)	26.2 (5.2)	27.1 (4.8)
	PH^j^ (reference range: 7-35)	17.8 (6.6)	19.9 (6.3)
	PS^k^ (reference range: 6-30)	18.6 (3.4)	20.1 (3.5)
	SR^l^ (reference range: 3-15)	9.8 (1.8)	9.4 (1.6)
**MoveMed fast tap test count, mean (SD)**			
	Dominant hand	24.7 (5.9)	24.7 (5.9)
	Nondominant hand	25.7 (8.3)	24.0 (7.9)
**MoveMed fast tap test intertap duration (seconds), mean (SD)**			
	Dominant hand	0.19 (0.12)	0.18 (0.10)
	Nondominant hand	0.15 (0.04)	0.15 (0.05)
**MoveMed hold test stability score (%), mean (SD)**			
	Dominant hand	83.3 (9.9)	72.1 (23.1)
	Nondominant hand	80.5 (15.7)	70.1 (25.7)
MoveMed typing test speed (keys per second), mean (SD)		1.60 (0.55)	1.55 (0.47)
MoveMed stand and walk test cadence (steps per minute), mean (SD)		59.6 (31.7)	64.6 (30.1)

^a^Participants were considered clinically stable if their P-mJOA scores at weeks 6 and 12 did not change by more than an MCID . Since the MCID is a range in degenerative cervical myelopathy (ie, 1-2 P-mJOA points), analyses were subgrouped into an MCID ≤1 group and an MCID ≤2 group to aid with the clinical interpretation of findings.

^b^MCID : minimum clinically important difference.

^c^P-mJOA: patient-derived modified Japanese Orthopaedic Association.

^d^MDUE: motor dysfunction of the upper extremity.

^e^MDLE: motor dysfunction of the lower extremity.

^f^SDUE: sensory dysfunction of the upper extremity.

^g^WHOQOL-Bref: World Health Organization Quality of Life Brief Version.

^h^QOL: quality of life.

^i^EH: environmental health.

^j^PH: physical health.

^k^PS: psychological health.

^l^SR: social relationship.

### Patient-Reported Outcomes

In both groups, participants averaged 60 (SD 15; MCID ≤2: mean 56.5, SD 12.4; MCID ≤1: mean 62.0, SD 18.4) years (Table 1). DCM severity ranged from mild to severe in MCID ≤2 (P-mJOA total score: 8-18) and from moderate to severe in MCID ≤1 (P-mJOA total score: 8-14). Upper limb motor function ranged from none to “unable to eat with spoon but able to move hands” in both groups (P-mJOA motor dysfunction of the upper extremity subscore: 2-5). Lower limb motor function ranged from none to “able to move legs but unable to walk” in MCID ≤2 (P-mJOA MDLE subscore: 2-7) and from “able to walk up and/or downstairs with an aid of a handrail” to “able to move legs but unable to walk” in MCID ≤1 (P-mJOA MDLE subscore: 2-4). In both groups, overall QOL perception ranged from “good” to “poor” (WHOQOL-Bref overall QOL: 2-4), while overall health perception ranged from “satisfied” to “dissatisfied” in MCID ≤2 (WHOQOL-Bref overall health: 2-4) and from “satisfied” to “neither poor nor good” in MCID ≤1 (WHOQOL-Bref overall health: 2-3).

### MoveMed and Tasks

On MoveMed, participants performed similarly across groups (Table 1). In the fast tap test, participants tapped the screen between 24 and 25 times and waited around 1500-1900 ms between taps on average. In the typing test, they typed around 1.5 keys per second. In the hold test, arm stability averaged 70% to 80% points, and in the stand and walk test, cadence averaged 60 steps per minute (SD 32).

### Reliability and Measurement Error

ICC of agreement coefficients for both groups are reported in Table 2. Coefficients for the hold and typing tests were ≥0.7 in both groups. Coefficients for the fast tap test count were also ≥0.7 in both groups. Coefficients for the fast tap test intertap duration, however, were ≥0.7 in the MCID ≤1 group only. Coefficients for the stand and walk test were 0.68 in MCID ≤1 and 0.62 in MCID ≤2. Higher coefficients were generally seen in the more conservative test-retest stability group (ie, MCID ≤1).

SEM of agreement and SDC of agreement for both groups are reported in Table 3. In MCID ≤1, SEM of agreement values for the fast tap, hold, typing, and stand and walk tests were ±1 tap, ±1%-3% points, ±0.06 keys per second, and ±10 steps per minute, respectively. In MCID ≤2, SEM of agreement values were ±1 tap, ±2%-4% stability score points, ±0.06 keys per second, and ±10 steps per minute, respectively. SDC of agreement values (ie, thresholds beyond which change scores can be considered genuine change and not change due to error [13]) were ±3 taps, ±4%-7% points, ±0.2 keys per second, and ±27 steps per minute, respectively, in MCID ≤1, and ±3-7 taps, ±7%-10% points, ±0.2 keys per second, and ±27 steps per minute in MCID ≤2, respectively. Lower measurement errors were generally seen in the more conservative MCID ≤1 group for the fast tap and hold tests.

**Table 2:** ICC^a^ of agreement coefficients for the MoveMed outcome measures.

Outcome measure and end point			Results, ICC	Participants, n (%)	Rating^b^	ROB^c^
**MCID^d^ ≤1**						
	**Fast tap test**					
		Count	DH^e^: 0.94; NH^f^: 0.92	5 (71)	DH: +; NH: +	No
		Intertap duration	DH: 0.97; NH: 0.84	5 (71)	DH: +; NH: +	No
	**Hold test**					
		Stability score	DH: 0.89; NH: 0.95	5 (71)	DH: +; NH: +	No
	**Typing test**					
		Speed	0.95	4 (57)	+	No
	**Stand and walk test**					
		Cadence	0.68	4 (57)	–	No
**MCID ≤2**						
	**Fast tap test**					
		Count	DH: 0.88; NH: 0.91	7 (100)	DH: +; NH: +	No
		Intertap duration	DH: 0.61; NH: 0.68	7 (100)	DH: –; NH: –	No
	**Hold test**					
		Stability score	DH: 0.77; NH: 0.75	7 (100)	DH: +; NH: +	No
	**Typing test**					
		Speed	0.98	6 (86)	+	No
	**Stand and walk test**					
		Cadence	0.62	5 (71)	–	No

^a^ICC: intraclass correlation.

^b^“+”=sufficient and “–”=insufficient.

^c^ROB: risk of bias.

^d^MCID : minimum clinically important difference.

^e^DH: dominant hand.

^f^NH: nondominant hand.

**Table 3:** Measurement error metrics for the MoveMed outcome measures.

Outcome measure and end point				SEM of agreement^a^		SDC of agreement^b^		Participants, n (%)
**MCID^c^ ≤1**								
	**Fast tap test**							
		Count	DH^d^: 0.9; NH^e^: 1.0		DH: 2.5; NH: 2.8		5 (71)	
		Intertap duration	DH: 0.02; NH: 0.0004		DH: 0.06; NH: 0.001		5 (71)	
	**Hold test**							
		Stability score	DH: 2.4; NH: 1.7		DH: 6.7; NH: 4.7		5 (71)	
	**Typing test**							
		Speed	0.06		0.2		4 (57)	
	**Stand and walk test**							
		Cadence	9.8		27.2		4 (57)	
**MCID ≤2**								
	**Fast tap test**							
		Count	DH: 0.80; NH: 0.91		DH: 2.2; NH: 6.6		7 (100)	
		Intertap duration	DH: 0.02; NH: 0.006		DH: 0.04; NH: 0.02		7 (100)	
	**Hold test**							
		Stability score	DH: 3.5; NH: 2.4		DH: 9.6; NH: 6.6		7 (100)	
	**Typing test**							
		Speed	0.06		0.2		6 (86)	
	**Stand and walk test**							
		Cadence	9.6		26.5		5 (71)	

^a^SEM: SE of the mean.

^b^SDC: smallest detectable change.

^c^MCID : minimum clinically important difference.

^d^DH: dominant hand.

^e^NH: nondominant hand.

### Risk of Bias Assessment

No risk of bias factors from the COSMIN Risk of Bias checklist were recorded, which was equivalent to a “very good” quality rating for methodological quality [13].

### Overall Assessment

Reliability ratings for both groups are also reported in Table 2. ICC of agreement coefficients were converted into ratings by comparing results to a critical threshold of 0.7. All unidimensional tests obtained sufficient ratings for test-retest reliability across groups. In MCID ≤1, sufficient ratings were obtained for the performance of the fast tap, hold, and typing tests. In MCID ≤2, sufficient ratings were obtained for the performance of the hold and typing tests and the total count of the fast tap test. The performance of the multidimensional stand and walk test was the only test that did not achieve a sufficient rating due to ICC of agreement coefficients of 0.68 in MCID ≤1 and 0.62 in MCID ≤2. Taken together, these data provide “very good” quality evidence for the overall reliability of the PerfOMs in the assessment of DCM.

## Discussion

### Principal Findings

The primary purpose of this study was to assess the reliability of MoveMed, a battery of PerfOMs in a mobile app, using primary and secondary outcomes following formal methods and criteria from the FDA and COSMIN. Two aspects were evaluated: test-retest reliability and measurement error. All PerfOMs demonstrated moderate to excellent test-retest reliability coefficients (Table 2) and low measurement errors (Table 3). These findings are important as they confirm that MoveMed’s PerfOs remain stable when DCM remains stable and provide reference values to aid researchers in discerning real versus random change for each PerfO in the future [13].

Few PerfOMs are investigated for reliability thoroughly, including smartphone apps [13,18-22]. Test-retest coefficients are often computed, but measurement errors are often ignored. This is detrimental to clinical practice as it leaves clinicians unable to confidently differentiate real versus random changes in outcomes. Furthermore, when reported in isolation, test-retest coefficients run the risk of optimistic interpretation. The basis for this is the truism in Portney [23] that “reliability cannot be interpret as an all-or-none condition [but rather as] attained to varying degrees.” This leads to a graded interpretation of coefficients on an (admittedly) arbitrary scale of “poor” (<0.50) to “moderate” (0.50-0.75) to “good” (>0.75) [23] and even “excellent” (>0.90) [24]. These criteria, however, do not help clinicians judge whether tools are “sufficiently” reliable for use or not. Consensus guidance has been developed for this purpose and recommends using an all-or-none cutoff of 0.70 [13,25]. In this study, all PerfOMs were deemed sufficiently reliable using this cutoff, bar the multidimensional stand and walk test, where the ICC of agreement coefficient was 0.68 in the conservative (MCID <1) group. We attribute this deficit primarily to the multidimensional nature of the construct, and optimization is underway to improve control of random error through greater standardization in an updated version of MoveMed, which will be released shortly.

This study also computed measurement errors, which enabled researchers to interpret readings [13]. Our findings suggest that changes beyond certain thresholds represent genuine variation in individual user performance rather than variation due to error. These thresholds are (1) changes above or below 7 taps in the fast tap test, (2) changes above or below 10 stability score points in the hold test, (3) changes above or below 0.2 keys per second in the typing test, (4) changes above or below 27 steps per minute in the stand and walk test (Table 3). A further study will be needed to elucidate the clinical significance of these thresholds (ie, what is the magnitude of change that should trigger a change in clinical care or is considered meaningful to the patient). This is referred to as the MCID and may be different from the SDC.

An important strength of this study is its design by individuals with formal training in clinimetrics. This is reflected in the absence of risk of bias factors from the COSMIN checklist in Table 2 and the study report. The use of the COSMIN manual is strongly encouraged by the authors, as its standards are considered to be a cornerstone in clinimetric validation and overlap importantly with industry guidance from the US FDA [26,27]. Another strength of this study was the use of PerfOMs that can collect several test-retest repeats quickly, ecologically, and longitudinally. This means that constructs should be captured more precisely, more reflective of pathology in the patient’s natural environment, and potentially more responsive to intervention.

Despite its conscientious design, this study has limitations. First, several participants were excluded from the analysis. Three quarters of the sample (n=20, 74%) were excluded due to the absence of follow-up questionnaire responses, and up to a tenth (n=3, 11%) due to missing repeated-measures data [17]. We attribute the former exclusion to the burden of repeating PROM completion for patients (via email) and the latter to heterogeneous task adherence. To maximize the clinical relevance of our findings, we stratified the included sample into demonstrably stable groups, namely, a conservative MCID ≤1 group and a less conservative MCID ≤2 group. This combination was chosen as the MCID of the mJOA is estimated to be between 1 and 2 due to variations in individual disease severity and methods of estimation [16]. The reliability was increased for the MCID ≤1 group compared to the MCID ≤2 group, further supporting the reliability of the measure itself. While MoveMed data were longitudinally available for 100% (N=27) of the sample, clinical stability could only be demonstrated in a subset, so it was deemed safer and more beneficial to report on the subset. In the next version of the app, in-app PROMs, reminders, and gamification will improve the homogeneity of adherence and follow-up. This should be advantageous for trials in the future, as it should decrease exclusion rates without compromising task or PROM prescription and, even, reduce the burden of PROM completion if the correlation to task performance is strong enough and can intermittently surrogate for it.

Second, given that self-reported outcome measures and data elements were used in a decentralized secondary setting, standards for patient-reported methods were adapted to assess performance-based methods. This was done to overcome the absence of standardized criteria in this field and because there is precedent for it in Terwee et al [18] and the COSMIN manual [13]. Third, people with a severe form of the disease may have been excluded from enrollment. This would be due to the exclusion of individuals who were unable to stand and walk without the assistance of another person. The potential risks of remote participation in this subset of individuals, however, were deemed to outweigh the benefits by the ethical committee. Further in-person research could address this limitation in the future.

### Conclusions

Software apps are increasingly being used to administer PerfOMs in medicine [28]. This study provides initial, “very good” quality evidence for the reliability of the smartphone-based battery of MoveMed PerfOMs in the context of adults living with DCM in the community. Most digital development to date has focused on running PerfOMs on wearable technology over smartphones. Smartwatches, for instance, have been frequently used to measure activity and movement, but test-retest reliability has been found to be insufficient at worst and variable at best [29,30].

Smartphone testing has recently emerged as a more reliable alternative [31,32]. In our experience, this is because smartphone testing (1) focuses on narrower constructs, (2) standardizes task performance (ie, conducts tasks in a controlled or stereotyped environment), and (3) controls for environmental variables better as a result. These principles incidentally reflect John Macleod’s fundamentals of good clinical examination [33]. Digital health technologies ought to embody these principles, just as clinicians do, if they hope to drive clinically meaningful and useful adoption in health care. The tests in this study show just one way in which these principles can be intentionally designed into deployment: we hope to see a more clinically driven design to “improve the reliability and precision of ... clinical assessment” [33] as new tools and technologies are released over the next few decades in health care.

## Abbreviations

COSMIN: Consensus-Based Standards for the Selection of Health Measurement Instruments
DCM: degenerative cervical myelopathy
FDA: Food and Drug Administration
ICC: intraclass correlation
ISO: International Organization for Standardization
MCID: minimum clinically important difference
MDLE: motor dysfunction of the lower extremity
mJOA: modified Japanese Orthopaedic Association
PerfO: performance-based outcome
PerfOM: performance-based outcome measure
P-mJOA: patient-derived modified Japanese Orthopaedic Association
PROM: patient-reported outcome measure
QOL: quality of life
SDC: smallest detectable change
SEM: SE of the mean
WHOQOL-Bref: World Health Organization Quality of Life Brief Version

## Data Availability

All data analyzed during this study are included in this published paper.
